# Supplementary material for: Iterative improvement in the automatic modular design of robot swarms
Source: PeerJ Comput Sci. 2020 Dec 7;6:e322. doi: 10.7717/peerj-cs.322 (PMC7924708; doi:10.7717/peerj-cs.322)
Supplement: Supplemental Information 3 [file peerj-cs-06-322-s003.zip › argos3/doc/api/standalone/a00584_source.html]

ARGoS: plugins/simulator/entities/proximity\_sensor\_equipped\_entity.cpp Source File


- Main Page
- Related Pages
- Namespaces
- Classes
- Files

- File List
- File Members

# plugins/simulator/entities/proximity\_sensor\_equipped\_entity.cpp

Go to the documentation of this file.

```
00001 
00007 #include "proximity_sensor_equipped_entity.h"
00008 #include <argos3/core/simulator/space/space.h>
00009 #include <argos3/core/simulator/entity/composable_entity.h>
00010 
00011 namespace argos {
00012 
00013    /****************************************/
00014    /****************************************/
00015 
00016    CProximitySensorEquippedEntity::SSensor::SSensor(const CVector3& c_offset,
00017                                                     const CVector3& c_direction,
00018                                                     Real f_range,
00019                                                     SAnchor& s_anchor) :
00020       Offset(c_offset),
00021       Direction(c_direction),
00022       Anchor(s_anchor) {
00023       Direction.Normalize();
00024       Direction *= f_range;
00025    }
00026 
00027    /****************************************/
00028    /****************************************/
00029 
00030    CProximitySensorEquippedEntity::CProximitySensorEquippedEntity(CComposableEntity* pc_parent) :
00031       CEntity(pc_parent) {
00032       Disable();
00033    }
00034    
00035    /****************************************/
00036    /****************************************/
00037 
00038    CProximitySensorEquippedEntity::CProximitySensorEquippedEntity(CComposableEntity* pc_parent,
00039                                                                   const std::string& str_id) :
00040       CEntity(pc_parent, str_id) {
00041       Disable();
00042    }
00043 
00044    /****************************************/
00045    /****************************************/
00046 
00047    CProximitySensorEquippedEntity::~CProximitySensorEquippedEntity() {
00048       while(! m_tSensors.empty()) {
00049          delete m_tSensors.back();
00050          m_tSensors.pop_back();
00051       }
00052    }
00053 
00054    /****************************************/
00055    /****************************************/
00056 
00057    void CProximitySensorEquippedEntity::Init(TConfigurationNode& t_tree) {
00058       try {
00059          /*
00060           * Parse basic entity stuff
00061           */
00062          CEntity::Init(t_tree);
00063          /*
00064           * Parse proximity sensors
00065           */
00066          /* Not adding any sensor is a fatal error */
00067          if(t_tree.NoChildren()) {
00068             THROW_ARGOSEXCEPTION("No sensors defined");
00069          }
00070          /* Go through children */
00071          TConfigurationNodeIterator it;
00072          for(it = it.begin(&t_tree); it != it.end(); ++it) {
00073             std::string strAnchorId;
00074             GetNodeAttribute(*it, "anchor", strAnchorId);
00075             /*
00076              * NOTE: here we get a reference to the embodied entity
00077              * This line works under the assumption that:
00078              * 1. the ProximitySensorEquippedEntity has a parent;
00079              * 2. the parent has a child whose id is "body"
00080              * 3. the "body" is an embodied entity
00081              * If any of the above is false, this line will bomb out.
00082              */
00083             CEmbodiedEntity& cBody = GetParent().GetComponent<CEmbodiedEntity>("body");
00084             if(it->Value() == "sensor") {
00085                CVector3 cOff, cDir;
00086                Real fRange;
00087                GetNodeAttribute(*it, "offset", cOff);
00088                GetNodeAttribute(*it, "direction", cDir);
00089                GetNodeAttribute(*it, "range", fRange);
00090                AddSensor(cOff, cDir, fRange, cBody.GetAnchor(strAnchorId));
00091             }
00092             else if(it->Value() == "ring") {
00093                CVector3 cRingCenter;
00094                GetNodeAttributeOrDefault(t_tree, "center", cRingCenter, cRingCenter);
00095                Real fRadius;
00096                GetNodeAttribute(t_tree, "radius", fRadius);
00097                CDegrees cRingStartAngleDegrees;
00098                GetNodeAttributeOrDefault(t_tree, "start_angle", cRingStartAngleDegrees, cRingStartAngleDegrees);
00099                CRadians cRingStartAngleRadians = ToRadians(cRingStartAngleDegrees);
00100                Real fRange;
00101                GetNodeAttribute(t_tree, "range", fRange);
00102                UInt32 unNumSensors;
00103                GetNodeAttribute(t_tree, "num_sensors", unNumSensors);
00104                AddSensorRing(cRingCenter,
00105                              fRadius,
00106                              cRingStartAngleRadians,
00107                              fRange,
00108                              unNumSensors,
00109                              cBody.GetAnchor(strAnchorId));
00110             }
00111             else {
00112                THROW_ARGOSEXCEPTION("Unrecognized tag \"" << it->Value() << "\"");
00113             }
00114          }
00115       }
00116       catch(CARGoSException& ex) {
00117          THROW_ARGOSEXCEPTION_NESTED("Initialization error in proximity sensor equipped entity", ex);
00118       }
00119    }
00120 
00121    /****************************************/
00122    /****************************************/
00123 
00124    void CProximitySensorEquippedEntity::Enable() {
00125       CEntity::Enable();
00126       for(size_t i = 0; i < m_tSensors.size(); ++i) {
00127          m_tSensors[i]->Anchor.Enable();
00128       }
00129    }
00130 
00131    /****************************************/
00132    /****************************************/
00133 
00134    void CProximitySensorEquippedEntity::Disable() {
00135       CEntity::Disable();
00136       for(size_t i = 0; i < m_tSensors.size(); ++i) {
00137          m_tSensors[i]->Anchor.Disable();
00138       }
00139    }
00140 
00141    /****************************************/
00142    /****************************************/
00143 
00144    void CProximitySensorEquippedEntity::AddSensor(const CVector3& c_offset,
00145                                                   const CVector3& c_direction,
00146                                                   Real f_range,
00147                                                   SAnchor& s_anchor) {
00148       m_tSensors.push_back(new SSensor(c_offset, c_direction, f_range, s_anchor));
00149    }
00150 
00151    /****************************************/
00152    /****************************************/
00153    
00154    void CProximitySensorEquippedEntity::AddSensorRing(const CVector3& c_center,
00155                                                       Real f_radius,
00156                                                       const CRadians& c_start_angle,
00157                                                       Real f_range,
00158                                                       UInt32 un_num_sensors,
00159                                                       SAnchor& s_anchor) {
00160       CRadians cSensorSpacing = CRadians::TWO_PI / un_num_sensors;
00161       CRadians cAngle;
00162       CVector3 cOff, cDir;
00163       for(UInt32 i = 0; i < un_num_sensors; ++i) {
00164          cAngle = c_start_angle + i * cSensorSpacing;
00165          cAngle.SignedNormalize();
00166          cOff.Set(f_radius, 0.0f, 0.0f);
00167          cOff.RotateZ(cAngle);
00168          cOff += c_center;
00169          cDir.Set(f_range, 0.0f, 0.0f);
00170          cDir.RotateZ(cAngle);
00171          AddSensor(cOff, cDir, f_range, s_anchor);
00172       }
00173    }
00174 
00175    /****************************************/
00176    /****************************************/
00177 
00178    void CProximitySensorEquippedEntity::AddSensorFan(const CVector3& c_center,
00179                                                      Real f_radius,
00180                                                      const CRadians& c_start_angle,
00181                                                      const CRadians& c_end_angle,
00182                                                      Real f_range,
00183                                                      UInt32 un_num_sensors,
00184                                                      SAnchor& s_anchor) {
00185       CRadians cSensorSpacing = (c_end_angle - c_start_angle) / (un_num_sensors - 1);
00186       CRadians cAngle;
00187       CVector3 cOff, cDir;
00188       for(UInt32 i = 0; i < un_num_sensors; ++i) {
00189          cAngle = c_start_angle + i * cSensorSpacing;
00190          cAngle.SignedNormalize();
00191          cOff.Set(f_radius, 0.0f, 0.0f);
00192          cOff.RotateZ(cAngle);
00193          cOff += c_center;
00194          cDir.Set(f_range, 0.0f, 0.0f);
00195          cDir.RotateZ(cAngle);
00196          AddSensor(cOff, cDir, f_range, s_anchor);
00197       }
00198    }
00199 
00200    /****************************************/
00201    /****************************************/
00202 
00203    REGISTER_STANDARD_SPACE_OPERATIONS_ON_ENTITY(CProximitySensorEquippedEntity);
00204    
00205    /****************************************/
00206    /****************************************/
00207 
00208 }
```

---

Generated on 10 Jul 2018 for ARGoS by 
 1.6.1
